# Asthmatic Bronchial Smooth Muscle Increases CCL5-Dependent Monocyte Migration in Response to Rhinovirus-Infected Epithelium

**DOI:** 10.3389/fimmu.2019.02998

**Published:** 2020-01-06

**Authors:** Benoit Allard, Hannah Levardon, Pauline Esteves, Alexis Celle, Elise Maurat, Matthieu Thumerel, Pierre Olivier Girodet, Thomas Trian, Patrick Berger

**Affiliations:** ^1^Univ-Bordeaux, Centre de Recherche Cardio-thoracique de Bordeaux, U1045, Département de Pharmacologie, CIC 1401, Bordeaux, France; ^2^INSERM, Centre de Recherche Cardio-thoracique de Bordeaux, U1045, CIC 1401, Bordeaux, France; ^3^CHU de Bordeaux, Service d'Exploration Fonctionnelle Respiratoire, Service de Chirurgie Thoracique, CIC 1401, Pessac, France

**Keywords:** mucosal immunology, bronchial remodeling, asthma, monocyte, rhinovirus, epithelium, smooth muscle, co-culture

## Abstract

Asthma exacerbations, a major concern in therapeutic strategies, are most commonly triggered by viral respiratory infections, particularly with human rhinovirus (HRV). Infection of bronchial epithelial (BE) cells by HRV triggers inflammation, notably monocyte recruitment. The increase of bronchial smooth muscle (BSM) mass in asthma, a hallmark of bronchial remodeling, is associated with the annual rate of exacerbations. The aim of the present study was to assess whether or not BSM could increase monocyte migration induced by HRV-infected BE. We used an advanced *in vitro* model of co-culture of human BE cells in air-liquid interface with human BSM cells from control and asthmatic patients. Inflammation triggered by HRV infection (HRV-16, MOI 0.1, 1 h) was assessed at 24 h with transcriptomic analysis and multiplex ELISA. *In vitro* CD14^+^ monocyte migration was evaluated with modified Boyden chamber. Results showed that HRV-induced monocyte migration was substantially increased in the co-culture model with asthmatic BSM, compared with control BSM. Furthermore, the well-known monocyte migration chemokine, CCL2, was not involved in this increased migration. However, we demonstrated that CCL5 was further increased in the asthmatic BSM co-culture and that anti-CCL5 blocking antibody significantly decreased monocyte migration induced by HRV-infected BE. Taken together, our findings highlight a new role of BSM cells in HRV-induced inflammation and provide new insights in mucosal immunology which may open new opportunities for prevention and/or treatment of asthma exacerbation.

## Introduction

Asthma is a chronic respiratory disease characterized by chronic inflammation, bronchial hyperresponsiveness and bronchial remodeling. Asthma exacerbations still represent a major concern in therapeutic strategies since they are characterized by an increase in symptoms and a decrease in lung function that is sufficient to require a change in treatment (1). Asthma exacerbations have been often associated with viral respiratory infections, with an estimated rate of 65–85% of all viral exacerbations in children and 50% in adults being caused by human rhinovirus (HRV) (2). HRV infection was largely restricted to the bronchial epithelium (BE) (3). HRV infection of BE triggered the release of a various range of mediators, such as antiviral interferons and pro-inflammatory cytokines (4). HRV-infected BE also produced chemokines, such as IL-25, IL-33, and thymic stromal lymphopoietin, therefore inducing immune cell migration toward lung tissue and subsequent inflammation (5).

HRV also presented the ability to enter and replicate in monocytes when they are co-cultured with BE cells (6). Monocytes are myeloid cells that give rise to macrophages, dendritic cells (7), and fibrocytes (8). While macrophage and dendritic cell involvement in asthma has been well-documented, the role of monocytes themselves has not been studied extensively, whereas several publications showed that they are recruited after viral- or bacterial-infection (9). Indeed, several chemokines have been shown to attract monocytes from blood circulation. CCL2 was considered as the main monocyte chemoattractant and has been shown to be associated with monocyte migration in many diseases. During viral- or bacterial-infection, increased amount of both CCL2 and CCL5 has been observed, in serum from asthmatic patients and these data correlate with those found in mouse models of asthma, in whom expression level of CCL2 in lung tissue and bronchoalveolar lavage fluids were also enhanced compared to control mice (10). In addition to CCL2, many other chemokines presented chemotactic properties on monocytes such as CCL3, CCL5, or CCL7 (9, 11). Importantly, monocyte may have an important role in HRV-induced exacerbation since sCD86, a pro-inflammatory mediator secreted by the intermediate monocyte subset, was highly expressed in serum of asthmatic patients under exacerbation (12).

An important feature of bronchial remodeling was the increase of bronchial smooth muscle (BSM) mass, which has been correlated with decreased lung function (13, 14). Interestingly, this enhanced BSM mass was also associated with an increased annual rate of exacerbations (15). Moreover, the reduction of BSM area by bronchial thermoplasty drastically decreased the rate of exacerbations (16, 17), but its mechanisms remain unknown. Surprisingly, the role of BSM on monocyte migration during exacerbation has never been explored.

Therefore, the goal of the present study was to assess whether or not BSM could increase monocyte migration induced by HRV-infected BE. The present study showed that HRV-induced monocyte migration was substantially increased in the co-culture of BE with asthmatic BSM, compared to that with control BSM. Furthermore, we determined that the major monocyte chemoattractant CCL2 was not involved but instead this increased migration was CCL5-dependent. Taken together, our findings highlight a new role of BSM cells in HRV-induced inflammation and provide new insight in mucosal immunology which may open new opportunities for the prevention and/or treatment of asthma exacerbation.

## Materials and Methods

### Study Populations

Patients with asthma were recruited from the “COBRA” cohort (“Cohorte Obstruction Bronchique et Asthme”; i.e., Bronchial Obstruction and Asthma Cohort) in the Clinical Investigation Center of Bordeaux (CIC, Hôpital Haut-Lévêque, Pessac, France) according to GINA (1). Non-asthmatic control subjects were recruited after surgical resection if they had normal lung function. Bronchial specimens from all subjects were obtained by either fiberoptic bronchoscopy or lobectomy, as previously described (18). All subjects gave their written informed consent to participate to the study after the nature of the procedure has been fully explained. The COBRA study received approval from the National Ethics Committee. Patients' characteristics are presented in Table 1.

**Table 1:** Patients' characteristics.

**Characteristics**	**Controls**	**Asthmatics**	***p*-value**
No. of patients	32	25	
Age, yr	64.48 ± 9.44	54.31 ± 18.19	0.08
Body mass index, kg/m^2^	25.34 ± 4.98	26.37 ± 5.98	0.72
**Treatments**			
LABA, No. of patients	0	24	
ICS, No. of patients	0	24	
OCS, No. of patients	0	5	
**FEV**_**1**_			
Liters	2.21 ± 0.49	2.11 ± 0.79	0.37
Percentage of predicted value	82.4 ± 24.23	78.93 ± 22.18	0.79
Percentage of FVC	72.45 ± 10.14	82.24 ± 15.77	0.04

### Cell Culture and Co-culture Model

Primary BSM cell culture was established, as described previously (19, 20). BSM cells (see subjects' characteristics Table S1) were only used from passages 2 to 5 to avoid BSM cell dedifferentiation into myofibroblasts or fibroblasts. Cell culture purity was assessed by immunocytochemistry using BSM-specific markers, with a requirement of αSMA- and calponin-positive cells ≥90%.

BE cells were obtained from surgical specimen of control subjects (see subjects' characteristics Table S2), as described previously (21). Briefly, BE cells were cultured in Pneumacult-Ex medium (Stemcell Technologies, Vancouver, Canada). After reaching 70% confluence, BE cells were grown on 0.4 μm pore-diameter insert with Pneumacult-ALI (complemented with hydrocortisone and heparin according to the manufacturer, Stemcell) and cultured in air-liquid interface (ALI) for 21 days, in order to obtain a fully differentiated BE. ALI-BE was either infected or not infected with HRV-16 (Gift from Dr. Brian Oliver, Woolcock Institute of Medical Research, Sydney, Australia) at a MOI of 0.1 for 1 h (100 μl of DMEM containing HRV-16, was dropped on the top of the insert and removed after 1 h). For HRV-16 infection, hydrocortisone was removed from Pneumacult-ALI medium. Supernatant were collected 24 h after the infection. The absence of HRV particles in the co-culture supernatant was confirmed by digital PCR (data not shown).

The co-culture was established by adding the ALI-BE insert to the BSM well for 1 week. Medium used for co-culture was a mix of that used for BSM and BE (50% of DMEM supplemented with 10% FBS and 50% of Pneumacult-ALI without hydrocortisone). The BE co-cultured with BSM were infected, as mentioned above for BE alone. The absence of HRV particles within BSM cells was confirmed by digital PCR (data not shown). Supernatant were collected from three independent experiments and stored at −80°C for further analysis. In total, 18 controls and 12 asthmatic BSM cells were co-cultured with at least three different BE cells.

### Rhinovirus Production and Infection

HRV-16 was used in this study since it represents the “major group” (which utilize the cell surface receptor intercellular adhesion molecule 1) and is used in a large number of experimental studies on human primary cells. The HRV-16 was propagated in HeLa cells with 2% serum, as previously described (22). HeLa cells were maintained in DMEM supplemented with 10% FBS. HRV-16 titration assay was established in Hela cells and digital PCR (Functional Genomic Centre of Bordeaux). The absence of any mycoplasma, bacterial and fungal contamination was confirmed by the use of PCR and bacterial/fungal cultures (Eurofins Genomics, Germany and Parasitology-Mycology department of Bordeaux University Health Centre, respectively).

### Monocyte Isolation

Primary monocytes were obtained from blood of asthmatic patients (see patients' characteristics Table S3). Briefly, peripheral blood mononuclear cells were separated from buffy coat using a Ficoll-gradient centrifugation method followed by a positive depletion with CD14 microbeads. Cells were then suspended at the adequate concentration in RPMI + Glutamax (Thermo Fisher Scientific, Waltham, Massachusetts, USA) supplemented with 8% FBS (Eurobio, Evry, France).

### Migration Assay

Primary monocyte migration was performed using transwell chambers (Thermo Fisher Scientific) with monocytes added to the upper chamber and culture supernatant to the lower chamber of 3.0 μm pore-diameter inserts, pre-coated with PLL(20)-g[3.5]-PEG(2) (SuSoS, Dübendorf, Switzerland) to prevent monocyte adhesion. The number of migrated cells was assessed after 4 h of migration by cell counting. The effect of chemokines on monocyte migration was assessed using neutralizing antibodies anti-CCL2 at 5 μg/ml (Biolegend, San Diego, CA) or anti-CCL5 at 1 μg/ml (R&D) antibodies, Armenian hamster IgG isotype control at 5 μg/ml (Biolegend) or mouse IgG1 isotype control at 1 μg/ml (Abcam, Cambridge, UK). For each experiment, results were first normalized with control medium (DMEM/ALI medium). Then, all the data were further normalized by the mean of the control condition (co-culture without HRV) and multiplied by 100 to display the results as percentage of migration.

Monocyte migration was also assessed using micro-optical coherence tomography (μ-OCT) (23). Briefly, it is a bio-imaging technic producing trans-sectional images based on the sample reflectance, with an axial and lateral resolution of 1 micron. μ-OCT measured variation of electric field amplitude of light scattered by the structure of the tissue. Primary BE cells seeded on 3 μm inverted transwell were placed in a customized holder specifically designed for transwells. The reconstituted BE was stimulated with TNF-α (100 ng/ml) 24 h before the migration assay. Cells were imaged using the μ-OCT imaging device in its inverted configuration and rotated around 10° to minimize direct reflection of the beam on flat surfaces. A 75 W lamp was circled with aluminum foil to transfer the heat and obtained a temperature around 37°C near the sample. 2D images were acquired by scanning the beam in a linear path (B-scan) over 1 mm length and 3D images were obtained by multiplying B-scan to scan 1 × 1 mm area. Time-lapsed acquisition was made by taking 512 two-dimensional sections every 10 min for 4 h. Typical Fourier-domain OCT reconstruction was applied to convert raw interferometric data to depth-resolved images that could be processed an analyzed in ImageJ to obtain 3D images.

### Multiplex Gene Expression Analysis

BE cells were lysed by adding 200 μl of lysis buffer RLT according to manufacturer's instruction (AllPrep® DNA/RNA/Protein Mini Kit, Qiagen, Hilden, Germany), 24 h after HRV infection. mRNA extracts were stored at −80°C and send to PARS-I (Plateforme Analytique de Recherche en Santé-Immunologie, Dr. Isabelle Pellegrin, Bordeaux, France) to be assessed with nCounter® FLEX using the inflammation panel (nanoString, Seattle, WA). Analysis was performed with nSolver™ Analysis software.

### Protein Expression

Protein expression of CCL2 and IL-6 was assessed by ELISA in supernatants (100 μl undiluted for controls and diluted 1/10 for HRV-16) following the manufacturer's instruction (Qiagen, Hilden, Germany). A custom Bio-Plex Assay (BioRad, Hercules, CA) was performed to assess chemokine expression in co-culture supernatants (50 μl undiluted) according to the manufacturer's instruction. Special plate reader (Bio-Plex MAGPIX™, BioRad) and software (Bio-Plex manager) were used. The assay running was based on the same principle as a classic ELISA, except that all the washing steps were made with a wash station (Bio-Rad), equipped with a magnetic field that kept the microbeads to the bottom of the well while performing washing steps.

### Statistical Analysis

All statistical tests were performed on Graphpad Prism 6 software (Graphpad Software, San Diego, CA). Results were displayed as mean ± SEM values of repeated independent experiments. Statistical tests used were ordinary one-way ANOVA with Newman-Keuls or Bonferonni's multiple comparisons test and Wilcoxon tests. Results were considered as statistically significant when *p* < 0.05.

## Results

### Enhanced Monocyte Migration Mediated by Rhinovirus-Infected BE

**Figure 1:**
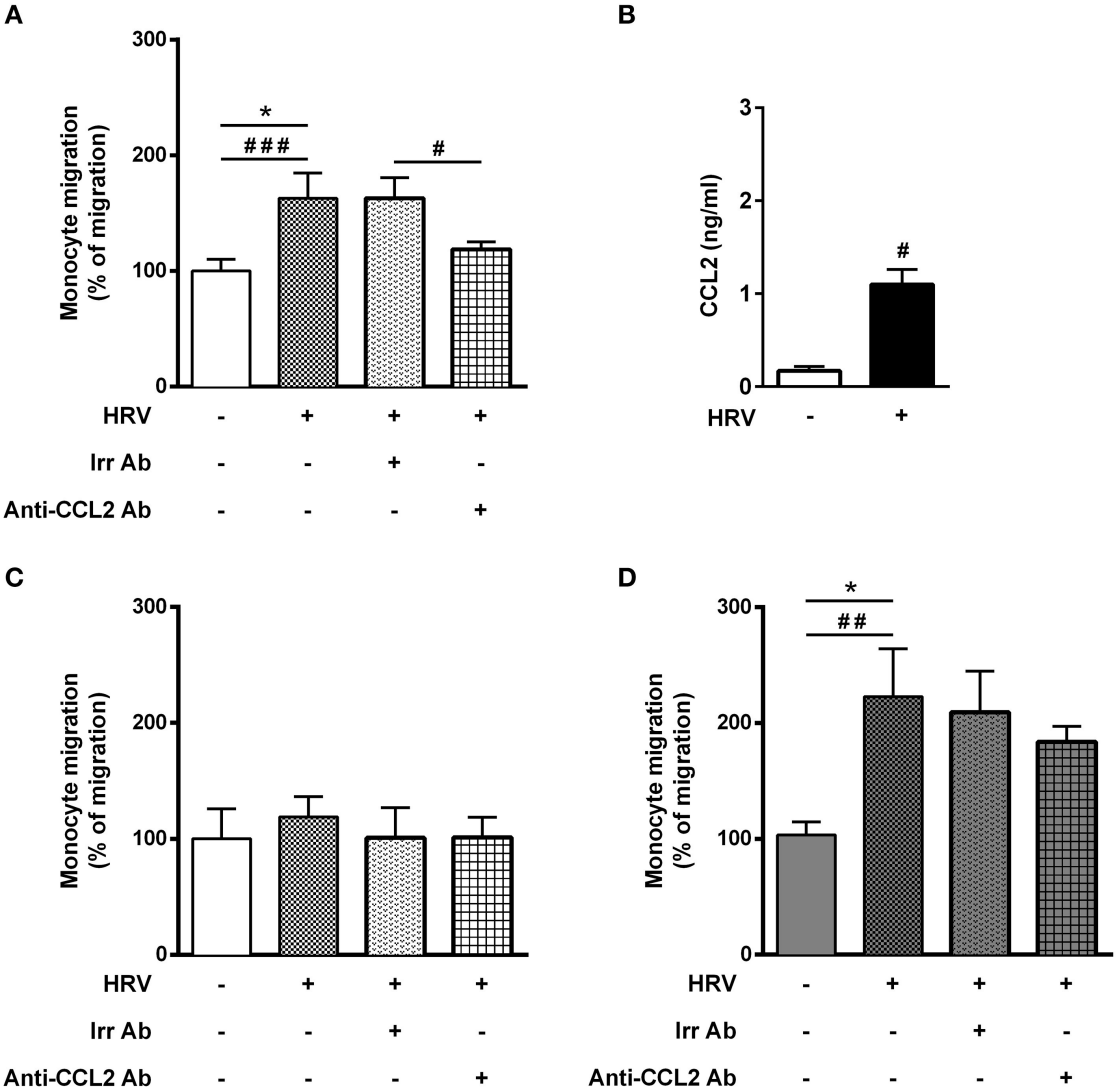
Asthmatic bronchial smooth muscle cell co-culture increases rhinovirus-mediated monocyte migration. **(A)** Monocyte migration was assessed in response to supernatants of reconstituted bronchial epithelial cells in air liquid interface infected or not with human rhinovirus (HRV-16; at MOI 0.1 for 1 h). The effect of CCL2 on rhinovirus-induced monocyte migration was evaluated by addition of blocking antibody (*n* = 7–11 per group). **(B)** CCL2 proteins were assessed from epithelial cell supernatant (*n* = 5 per group). **(C)** Monocyte migration was assessed in response to supernatants of reconstituted bronchial epithelial cells in air liquid interface co-cultured for 1 week with bronchial smooth muscle cells from control (*n* = 5–9 per group) or **(D)** asthmatic patients (*n* = 5–8 per group). Data are presented as mean ± SEM values of three independent experiments (Wilcoxon test, ^#^*P* < 0.05; ^##^*P* < 0.01; ^###^*P* < 0.001 and ordinary one way anova, Bonferroni's multiple comparisons test, *P < 0.05 compare the mean of HRV+ alone with the mean of every other columns).

Since BE is the first line of defense against respiratory viruses, we first sought to assess monocyte migration in response to supernatant of HRV-infected BE cells alone, cultured in ALI. As anticipated, a significant increase of monocyte migration (62%) was observed with HRV-infected BE supernatant (Figure 1A). Since HRV infection of BE cells induce CCL2 production (24), the major monocyte chemoattractant protein, we assessed CCL2 mRNA and protein levels in BE cell lysates. Although there was no difference in mRNA level at 24 h (data not shown) a significant increase of CCL2 protein was observed in HRV-infected BE (Figure 1B). We then used an anti-CCL2 neutralizing antibody to confirm that HRV-mediated monocyte migration was dependent on CCL2. As expected, this antibody virtually abrogated monocyte migration (Figure 1A).

We further assessed whether monocyte may cross the ALI-BE barrier. To that extent, we designed an inverted model with BE cells seeded on the inverted side of the insert of the transwell and we used an advanced system of OCT-imaging (μOCT), to perform live-imaging of inflammatory cell migration for 4 h (Figure S1). While neutrophil trans-epithelial migration could be demonstrated (Figure S1B), monocytes migration was not observed within the time of the experiment (Figure S1C). Thus, all subsequent migration assays were then performed using the modified Boyden Chamber.

### Asthmatic Bronchial Smooth Muscle Co-culture Increased Rhinovirus-Mediated Monocyte Migration

**Figure 2:**
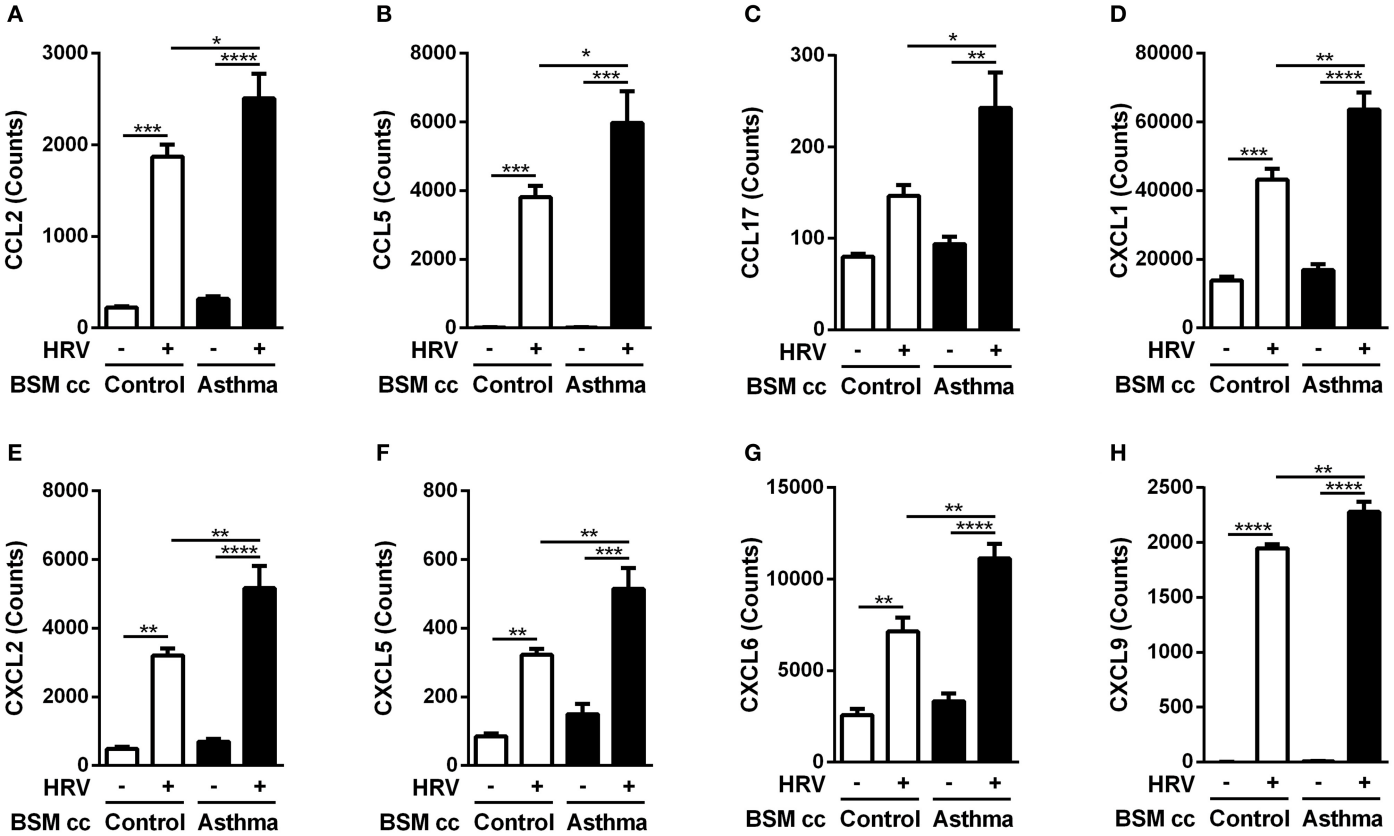
Asthmatic bronchial smooth muscle cell co-culture increases chemokines gene expression in bronchial epithelial cells after rhinovirus infection. **(A)** CCL2, **(B)** CCL5, **(C)** CCL17, **(D)** CXCL1, **(E)** CXCL2, **(F)** CXCL5, **(G)** CXCL6, **(H)** CXCL9 mRNA were quantified in epithelial cells by multiplex gene expression analysis. Data are presented as mean ± SEM values (*n* = 3 per group, one-way ANOVA, Newman-Keuls multiple comparisons test, *P < 0.05; **P < 0.01; ***P < 0.001; ****P < 0.0001).

Surprisingly, HRV infection of BE co-cultured with control BSM did not increase monocyte migration (Figure 1C). By contrast, a significant 2-fold increased migration was observed in HRV-infected BE when co-cultured with BSM from asthmatic patients (Figure 1D). Moreover, this migration was not related to CCL2, since the use of an anti-CCL2 neutralizing antibody did not alter monocyte migration, suggesting the involvement of other chemokines (Figure 1D). Importantly, no HRV-16 particle has been detected in both co-culture supernatant and BSM cells (data not shown) suggesting minor modification of epithelium integrity. In order to identify potential monocyte chemoattractants in this co-culture model, we performed a transcriptomic analysis of BE cell lysates from the different co-culture conditions. Several chemokines emerged as potential actors for monocyte migration since they presented mRNA levels significantly increased in HRV-infected BE co-cultured with asthmatic BSM compared to that co-cultured with control BSM: CCL2, CCL5, CCL17, CXCL1, CXCL2, CXCL5, CXCL6, and CXCL9 (Figure 2). Whereas both CCL4 and CCL23 mRNA levels were also increased in HRV-infected BE co-cultured with asthmatic BSM compared to that co-cultured with control BSM, the absolute counts were too low to be considered as pertinent (Figure S2). Additional chemokine transcriptomic levels were also measured but did not present any significant difference between HRV-infected BE co-cultured with control vs. asthmatic BSM (Figure S2). Transcriptomic analyses also demonstrated an increased expression of genes involved in the pro-inflammatory response, such as IL-1A, IL-6, or TNF-α (Figure S3A), as well as HRV-induced genes, like IFNA1, IFNB1, IFIT1, IFIT3, which all belonged to the interferon pathway (Figure S3B).

**Figure 3:**
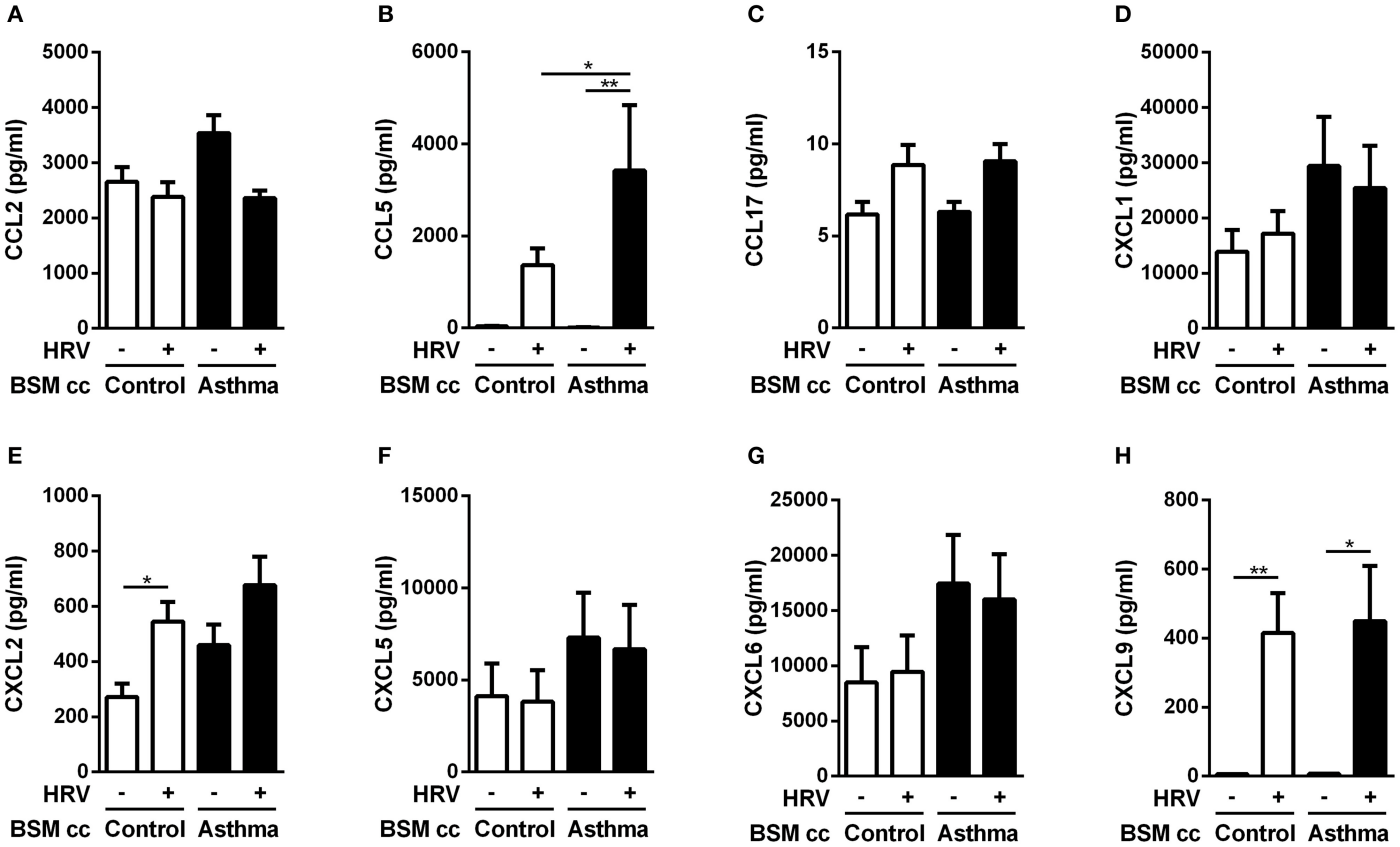
Asthmatic bronchial smooth muscle cell co-culture increases CCL5 expression in epithelial cells after rhinovirus infection. **(A)** CCL2, **(B)** CCL5, **(C)** CCL17, **(D)** CXCL1, **(E)** CXCL2, **(F)** CXCL5, **(G)** CXCL6, **(H)** CXCL9 proteins were quantified in co-culture supernatants by multiplex ELISA. Data are presented as mean ± SEM values (*n* = 8–11 per group, one-way ANOVA, Newman-Keuls multiple comparisons test, *P < 0.05; **P < 0.01).

The protein level of the chemokines of interest was then measured using multiplex ELISA assay (Figures 3A–H). Since anti-CCL2 blocking antibody was unable to alter monocyte migration induced by HRV-infected BE co-cultured with asthmatic BSM (Figure 1D), it was not surprising to identify no significant difference in CCL2 protein expression (Figure 3A). From the eight targets pre-selected from the transcriptomic analysis, only CCL5 presented a differential protein expression between HRV-infected BE co-cultured with asthmatic BSM compared to that co-cultured with control BSM (Figure 3B).

### Enhanced Monocyte Migration Was CCL5-Dependent

**Figure 4:**
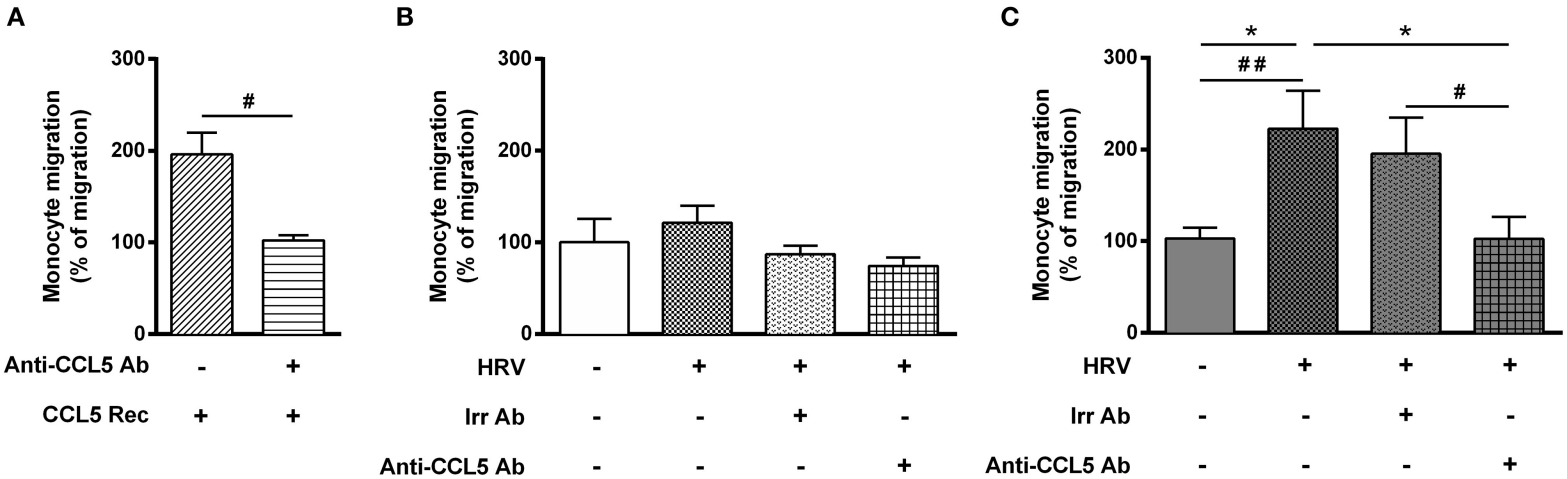
Increase rhinovirus-mediated monocyte migration induced by asthmatic bronchial smooth muscle is CCL5 dependent. Monocyte migration was assessed in response to recombinant CCL5 (CCL5 Rec) **(A)** as a positive control, or to supernatants of reconstituted bronchial epithelial cells in air liquid interface co-cultured with bronchial smooth muscle cells from **(B)** control (*n* = 6–9 per group) or **(C)** asthmatic patients (*n* = 6–8 per group). The effect of CCL5 on rhinovirus-induced monocyte migration was evaluated by addition of blocking antibody (Anti-CCL5 Ab). Data are presented as mean ± SEM values of three independent experiments (Wilcoxon test, ^#^*P* < 0.05; ^##^*P* < 0.01 and ordinary one way anova, Bonferroni's multiple comparisons test, *P < 0.05 compare the mean of HRV+ alone with the mean of every other columns).

To finally assess the role of CCL5 on monocyte migration, we performed additional migration experiments. First, adding recombinant CCL5 to non-infected-BE cell medium increased monocyte migration, which was abolished using an anti-CCL5 neutralizing antibody (Figure 4A). Second, anti-CCL5 neutralizing antibody did not decrease monocyte migration in HRV-infected-BE co-cultured with control BSM cells (Figure 4B), whereas it abolished the increased monocyte migration in HRV-infected-BE co-cultured with asthmatic BSM cells (Figure 4C).

## Discussion

Taken together, these results showed that the BSM from asthmatic patients increased the effects of HRV infection of the BE in terms of both pro-inflammatory response and monocyte migration in a CCL5-dependent manner.

In the present study, we focused our attention on the effects of HRV infected-BE on monocytes migration. Indeed, monocytes are recruited in the bronchial wall of asthmatic patients after infection (25, 26). Moreover, Shi et al. found that sCD86, a pro-inflammatory mediator secreted by the intermediate monocyte subset, was highly expressed in serum of asthmatic patients under exacerbation (12). In addition, monocyte-derived dendritic cells are sufficient and necessary to induce airway inflammation in mouse models of asthma (27, 28). In a mouse model of allergic airway inflammation using cockroach antigen, the increased monocytes/macrophage infiltration was related to subepithelial accumulation of versican and hyaluronan, two important proteins from the extracellular matrix involved in airway remodeling (29).

These results demonstrated the interest of using an advanced model of co-culture, since the crosstalk between BSM and BE cells altered the “classical” pattern of monocyte migration. Indeed, BSM can produce chemotactic proteins and therefore attract many inflammatory cells. In the context of asthma, BSM may attract mast cells (30), as well as T cells (14). However, to the best of our knowledge, there is no data regarding the attraction of monocytes by BSM cells. Using the co-culture model with asthmatic BSM cells, blocking CCL2 did not inhibit monocyte migration, suggesting a role of other chemokines in monocyte migration. Instead, transcriptomic and protein analyses showed an increased expression of CCL5, and functional migration assay demonstrated that the migration was CCL5-dependent. CCL5 can bind the chemokine receptors CCR1, CCR3, and CCR5 and monocytes express CCR1 and CCR5 on their surface (31). Whether CCL5/CCR1 axis was more important than CCL5/CCR5 axis in monocytes recruitment was not investigated in the present study. CCL5 is a potent chemoattractant for monocytes, T helper cells and eosinophils (32–34). Using a similar co-culture model, Malavia et al. showed that secreted mediators, such as IL-8, IL-6, or CCL2, were enhanced, especially when BE was mechanically injured with a pipette tip to mimic epithelial disruption (35). Moreover, HRV-induced CCL5 production by BE can also induce BSM cell chemotaxis (36). However, all these experiments were performed only with non-asthmatic BSM cells. Therefore, our own co-culture model with asthmatic BSM on BE chemokine expression and monocyte migration provided new insights regarding the role of asthmatic BSM in HRV-induced inflammation. Surprisingly, the co-culture system with control BSM abrogated the monocyte migration induced by HRV infection. These results suggest that a crosstalk between BE and BSM in healthy subjects could moderate inflammatory signaling in response to HRV infection whereas asthmatic BSM altered this crosstalk toward a pro-inflammatory response. Indeed, HRV-infection of BE co-cultured with asthmatic BSM increased the production of pro-inflammatory mediators (IL-1A, IL-6, TNF-alpha) at the transcriptional level compared to that co-cultured with control BSM cells. Moreover, HRV-infected BE-co-cultured with asthmatic BSM also increased IL-8 and IL-15 levels. By contrast, such an experimental condition did not alter type 2 inflammation (i.e., IL-4, IL-5).

In this study, we showed that CCL2 remained the main monocyte chemoattractant upon HRV infection when BE was cultured alone in ALI. CCL2 was considered as the main monocyte chemoattractant and was produced by many cell types, including BSM (37). Constitutive or stimulated CCL2 secretion by human asthmatic BSM has already been shown *in vitro* (38). BE cells were also able to produce CCL2 in response to other respiratory virus infection (39). However, *in vitro*, no effect of HRV-16 infection of BE on CCL2 production could be found in the literature. For instance, Keininger et al. did not observe any difference in CCL2 expression after HRV-16 infection, unlike infection with HRV-1B (40). These contradictory findings might be related to the infection protocol and the multiplicity of infection used. In addition, some of these studies used a monolayer of BE cells grown in liquid phase and not a pseudostratified BE grown in ALI (41). Similarly, it has been previously shown that HRV infection of such a monolayer of primary BE cells induced CCL5 expression (42), whereas in our hands, BE alone cultured in ALI produced CCL2. It is also important to mention that the HRV-related increased migration was not related to lower BE junction, since no HRV particle was observed in both co-culture supernatants and BSM cells. Moreover, the inflammatory response of BE was only due to HRV-16 infection since UV-inactivated HRV-16 did not trigger IL-6 production (Figure S4).

Several limitations can be discussed in this study. Firstly, BE cells were only obtained from control patients. Since the goal of the present study was to assess the role of BSM on monocyte migration induced by HRV-infected BE, we thus compared asthmatic and control BSM in a paired fashion. Moreover, when we previously evaluated the effects of BE on BSM cells, results were similar when asthmatic and control BE cells were used (21). Furthermore, the barrier function of the BE is compromised in asthma, which would improve the passage of viruses across the BE (43). Secondly, this study used primary cells, which are complex to obtain from patients and explain why we limited the study to a low number of patients. One can argue that we are not powered enough for non-significant results. Thirdly, we only used monocyte from asthmatic patients. Indeed, the current ethic protocol did not plan to use blood from control subjects. It has been shown that intermediate monocytes CD14^hi^ CD16^+^ were increased in severe asthmatic patients (44) which may impact the migration pattern of monocytes, as well as the differential chemokine receptor expression pattern. Fourth, we provided a mechanistic explanation for monocyte migration but further studies are required to understand how CCL5 production is increased in the asthmatic condition. Finally, our *in vitro* findings indicated that CCL5 may play a role in monocyte recruitment in rhinovirus-induced asthma exacerbation. This has to be confirmed with further *in vivo* or *ex vivo* studies.

In conclusion, this study highlighted a new role of BSM in asthma which altered BE response against HRV infection. These results were in agreement with the double association of, on the one hand, the increased exacerbation rate in asthmatic patients with increased BSM mass (15), and, on the other hand, the decreased exacerbation rate induced by bronchial thermoplasty, which decreased BSM mass (16, 17). However, whether or not BSM was directly involved in HRV-induced asthma exacerbation remained to be further elucidated.

## Data Availability Statement

All datasets generated for this study are included in the article/Supplementary Material.

## Ethics Statement

The studies involving human participants were reviewed and approved by the National Ethics Committee. The patients/participants provided their written informed consent to participate in this study.

## Author Contributions

BA and HL designed the research, performed the experiments, collected, analyzed and interpreted the data, and wrote the manuscript. PE, AC, and EM performed the experiments, analyzed the data, and revised the final manuscript. MT and PG provided the human samples from the Clinical Investigation Center of Bordeaux and revised the final manuscript. TT and PB designed the research, supervised the study, analyzed the data, and revised the manuscript.

### Conflict of Interest

The authors declare that the research was conducted in the absence of any commercial or financial relationships that could be construed as a potential conflict of interest.

## Supplementary Material

Figure S1 Monocyte do not cross epithelium in a short migration assay. Illustration of monocyte migration through a reconstituted bronchial epithelium evaluated by micro-OCT. **(A)** At T 0 min, the epithelium is inverted and neutrophils (white arrows) **(B)** or monocytes **(C)** are added on the upper chamber and set to migrate for 4 h. (Scale bar = 100 μm).

Figure S2 Chemokine expression in co-cultured epithelial cells after rhinovirus infection. Additional chemokines mRNA were quantified in epithelial cells by multiplex gene expression analysis. Data are presented as mean ± SEM values (*n* = 3 per group, one-way ANOVA, Newman-Keuls multiple comparisons test, ^*^*P* < 0.05; ^**^*P* < 0.01; ^***^*P* < 0.001; ^****^*P* < 0.0001).

Figure S3 Asthmatic bronchial smooth muscle cell co-culture increases inflammation in epithelial cells after rhinovirus infection. **(A)** Cytokines and **(B)** rhinovirus-mediated genes were assessed in epithelial cells by multiplex gene expression analysis. Data are presented as mean ± SEM values (*n* = 3 per group, one-way ANOVA, Newman-Keuls multiple comparisons test, ^*^*P* < 0.05; ^**^*P* < 0.01; ^***^*P* < 0.001; ^****^*P* < 0.0001).

Figure S4 Human rhinovirus infection induces IL-6 expression by bronchial epithelial cells. IL-6 was quantified in supernatants of BE cells infected by HRV or UV-inactivated HRV. Data are presented as mean ± SEM values (*n* = 6 per group, one-way ANOVA, Bonferroni's multiple comparisons test, ^***^*P* < 0.001).

Table S1 Patients' characteristics for BSM.

Table S2 Control subjects' characteristics for BE.

Table S3 Asthmatic patients' characteristics for blood.
