# Supplementary figures and images for: Asthmatic Bronchial Smooth Muscle Increases CCL5-Dependent Monocyte Migration in Response to Rhinovirus-Infected Epithelium
Source: Front Immunol. 2020 Jan 6;10:2998. doi: 10.3389/fimmu.2019.02998 (PMC6956660; doi:10.3389/fimmu.2019.02998)

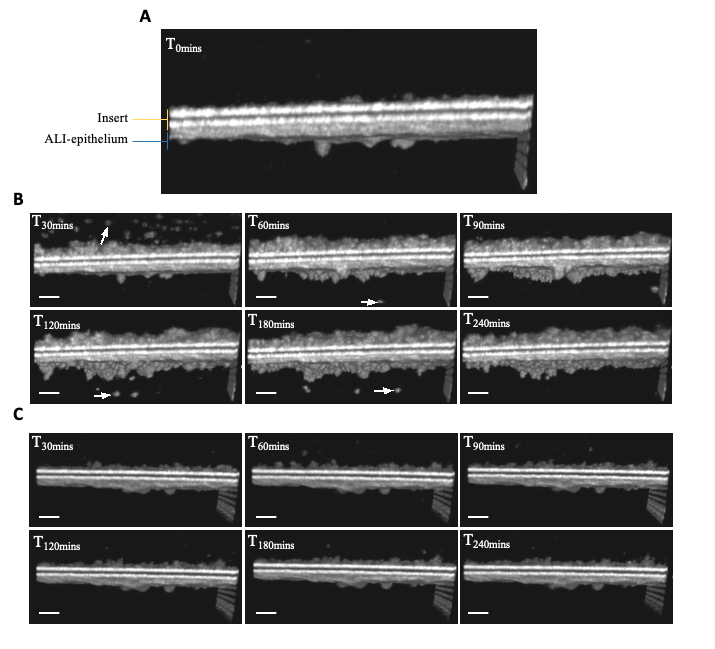

Supplement: Figure S1 — Monocyte do not cross epithelium in a short migration assay. Illustration of monocyte migration through a reconstituted bronchial epithelium evaluated by micro-OCT. (A) At T 0 min, the epithelium is inverted and neutrophils (white arrows) (B) or monocytes (C) are added on the upper chamber and set to migrate for 4 h. (Scale bar = 100 μm). [file Image_1.TIF]

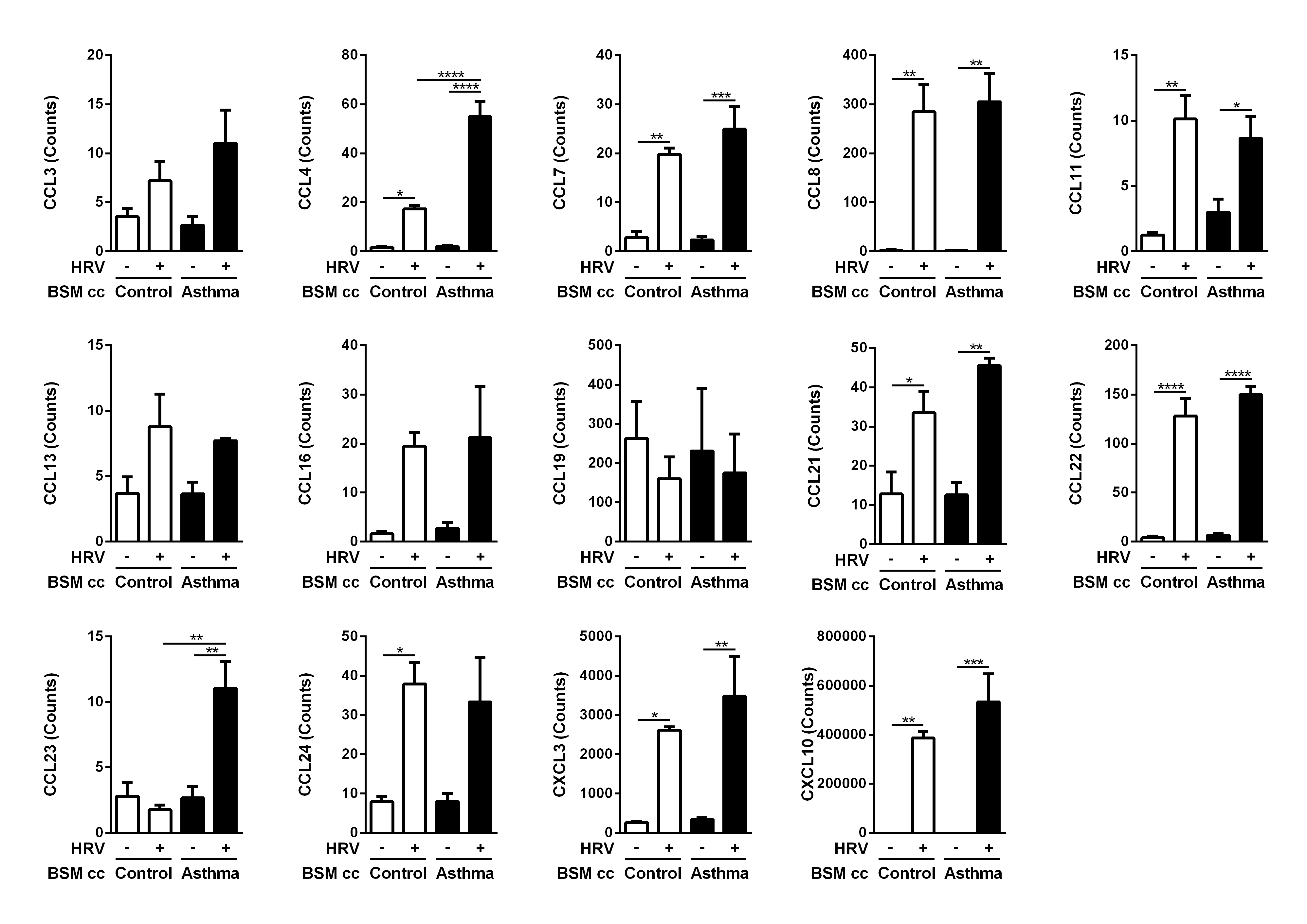

Supplement: Figure S2 — Chemokine expression in co-cultured epithelial cells after rhinovirus infection. Additional chemokines mRNA were quantified in epithelial cells by multiplex gene expression analysis. Data are presented as mean ± SEM values (n = 3 per group, one-way ANOVA, Newman-Keuls multiple comparisons test, *P < 0.05; **P < 0.01; ***P < 0.001; ****P < 0.0001). [file Image_2.TIF]

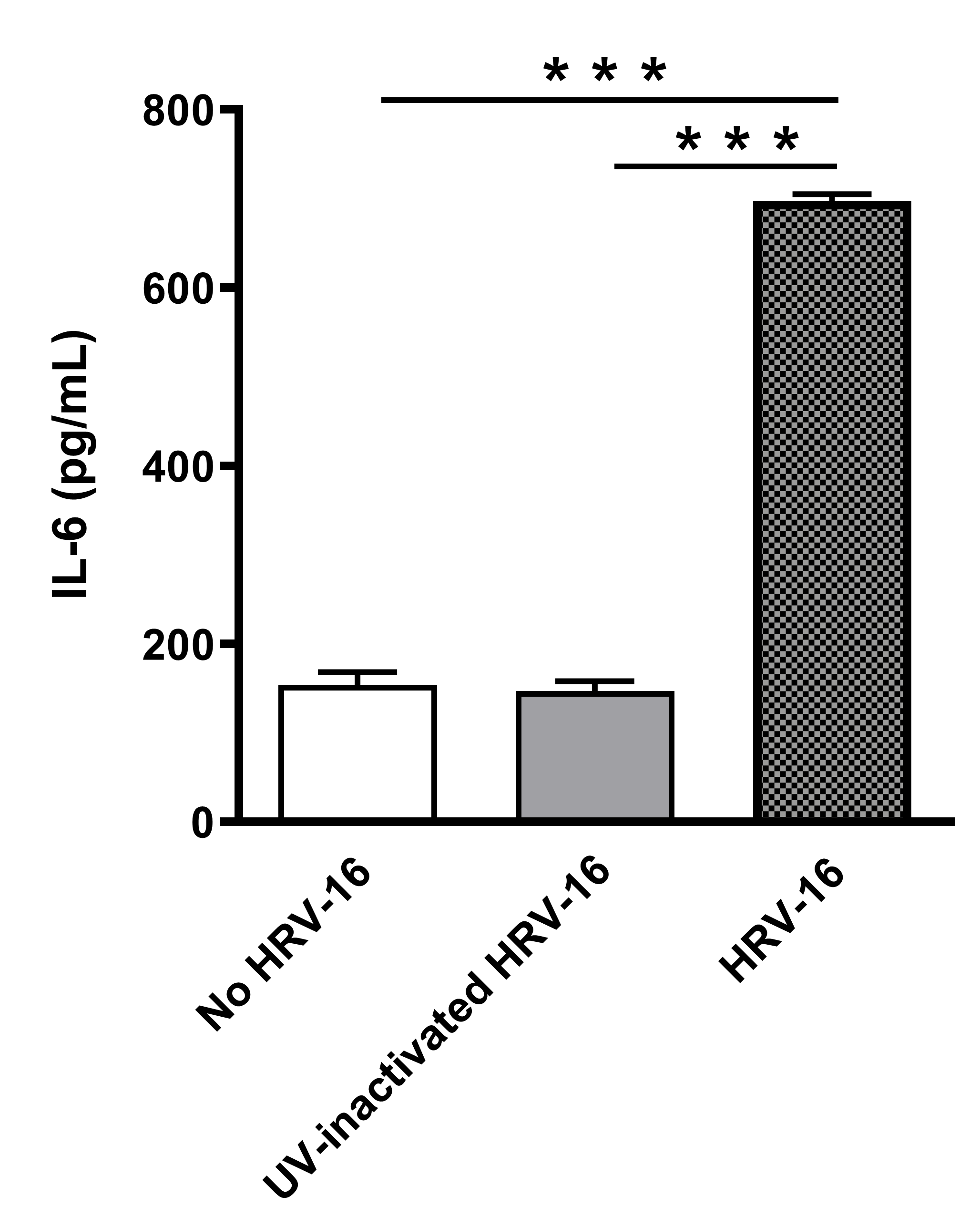

Supplement: Figure S4 — Human rhinovirus infection induces IL-6 expression by bronchial epithelial cells. IL-6 was quantified in supernatants of BE cells infected by HRV or UV-inactivated HRV. Data are presented as mean ± SEM values (n = 6 per group, one-way ANOVA, Bonferroni's multiple comparisons test, ***P < 0.001). [file Image_4.TIF]
