# Supplementary material for: Asthmatic Bronchial Smooth Muscle Increases CCL5-Dependent Monocyte Migration in Response to Rhinovirus-Infected Epithelium
Source: Front Immunol. 2020 Jan 6;10:2998. doi: 10.3389/fimmu.2019.02998 (PMC6956660; doi:10.3389/fimmu.2019.02998)
Supplement: Table S1 — Patients' characteristics for BSM. [file Data_Sheet_1.docx]

| Characteristics | Controls | Asthmatics | p value |
| --- | --- | --- | --- |
| No. of patients | 18 | 12 |  |
| Age, yr | 64 ± 2.03 | 55 ± 5.38 | 0.29 |
| Body mass index, kg/m² | 25.22 ± 1.10 | 27.24 ± 1.76 | 0.59 |
| **Treatments** |  |  |  |
| LABA, no. of patients | 0 | 12 |  |
| ICS, no. of patients | 0 | 12 |  |
| OCS, no. of patients | 0 | 2 |  |
| **FEV1** |  |  |  |
| Liters | 2.16 ± 0.12 | 2.02 ± 0.24 | 0.39 |
| Percentage of predicted value | 82.41 ± 6.39 | 80.51 ± 8.15 | 0.85 |
| Percentage of FVC | 70 ± 10.58 | 72 ± 10.31 | 0.68 |

**Table S1: Patients’ characteristics for BSM**

| Characteristics | Epithelium |
| --- | --- |
| No. of subjects | 14 |
| Age, yr | 64 ± 3.35 |
| Body mass index, kg/m² | 25.63 ± 1.69 |
| **Treatments** |  |
| LABA, no. of patients | 0 |
| ICS, no. of patients | 0 |
| OCS, no. of patients | 0 |
| **FEV1** |  |
| Liters | 2.3 ± 0.16 |
| Percentage of predicted value | 82.4 ± 7.31 |
| Percentage of FVC | 74 ± 10.25 |

**Table S2: Control subjects’ characteristics for BE**

| Characteristics | Blood Monocytes |
| --- | --- |
| No. of patients | 13 |
| Age, yr | 57.22 ± 4.30 |
| Body mass index, kg/m² | 27.21 ± 1.86 |
| **Treatments** |  |
| LABA, no. of patients | 12 |
| ICS, no. of patients | 12 |
| OCS, no. of patients | 3 |
| **FEV1** |  |
| Liters | 2.243 ± 0.25 |
| Percentage of predicted value | 76.83 ± 3.74 |
| Percentage of FVC | 69 ± 4.14 |

**Table S3: Asthmatic patients’ characteristics for bloodAuthor contributions statement**

BA and HL designed the research, performed experiments, collected, analyzed, and interpreted data and wrote the manuscript. PE, AC and EM performed experiments and analyzed data and revised the final manuscript. MT and POG provided human samples from the Clinical Investigation Center of Bordeaux and revised the final manuscript. TT and PB designed the research, supervised the study, analyzed data, and revised the manuscript.

**Conflict of interest statement**

The authors declare that the research was conducted in the absence of any commercial or financial relationships that could be construed as a potential conflict of interest.
